# Supplementary material for: Kindlin-2 haploinsufficiency protects against fatty liver by targeting Foxo1 in mice
Source: Nat Commun. 2022 Feb 23;13:1025. doi: 10.1038/s41467-022-28692-z (PMC8866405; doi:10.1038/s41467-022-28692-z)
Supplement: Supplementary file 1 — Supplementary Information [file 41467_2022_28692_MOESM1_ESM.pdf]

Kindlin-2 insufficiency protects against NAFLD by targeting FOXO1 in mice

Gao et.al

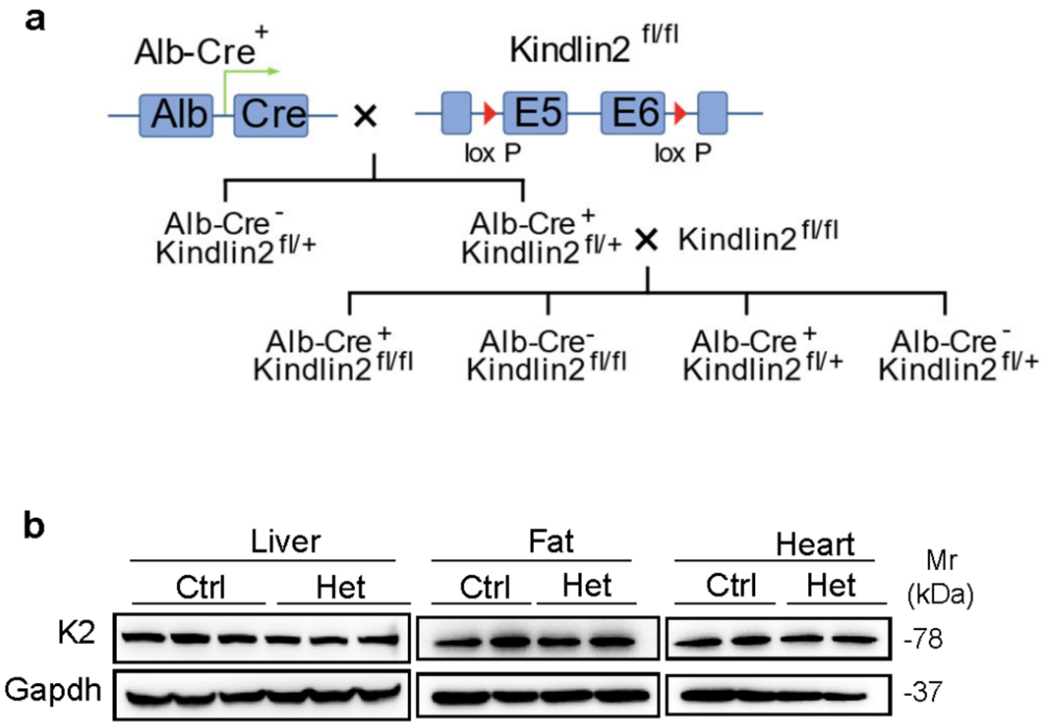

Supplementary Figure 1. Breeding strategy and PCR genotyping. a Breeding strategy to generate the Alb-Cre*; Kindlin-2*$^{fl/+}$ (Het) mice and other genotypes. **b** Expression of Kindlin-2 (K2) protein in different organs. Kindlin-2 expression after HFD feeding was examined by western blotting. Protein extracts (20 µg) were used for western blotting from each sample. Gapdh was used as a loading control. **b** Data are representative of three biologically independent replicates.

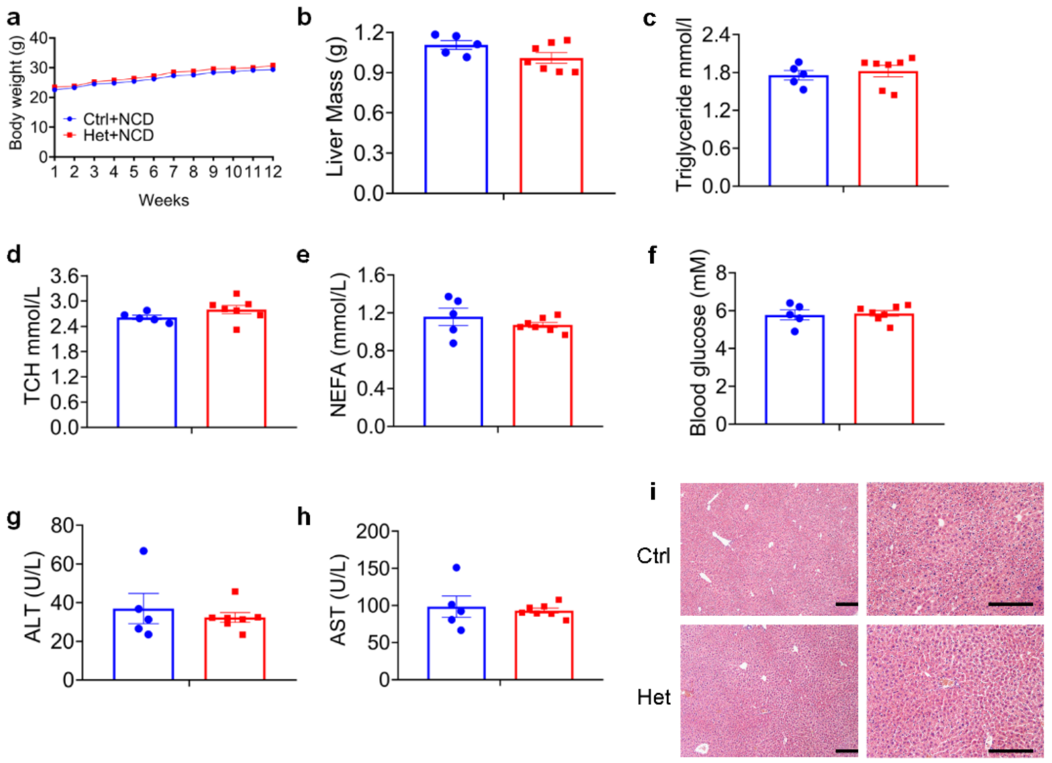

Supplementary Figure 2. Effects of Kindlin-2 haploinsufficiency in hepatocyte on the body weight, liver weight, lipid metabolism, and liver function in mice under NCD. a Six-week-old control and Het male mice were fed on NCD for 12 weeks; body weight was recorded every week. (*n* = 5 for the Ctrl group and *n* = 7 for the Het group) **b** Liver mass. (*n* = 5 for the Ctrl group and *n* = 7 for the Het group) **c**-**f** Serum TG, TCH, NEFA and blood glucose levels. (*n* = 5 for the Ctrl group and *n* = 7 for the Het group) **g**, **h** Serum ALT and AST levels. (*n* = 5 for the Ctrl group and *n* = 7 for the Het group) **i** H/E staining. Representative H/E staining images of liver sections are shown. Scale bar: 100 μm. **b-h** Data are presented as mean ± SEM. **i** Data are representative of three biologically independent replicates.

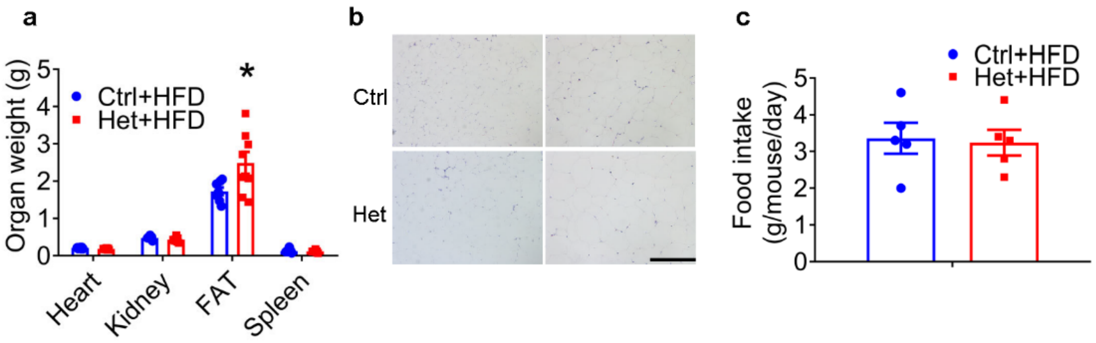

Supplementary Figure 3. Effects of Kindlin-2 haploinsufficiency in hepatocyte on adipose tissue mass, histology and food intake under HFD condition. a Organs weight after 12 weeks HFD feeding. ($n = 8$) **b** H/E staining. Representative H/E staining images of white adipose tissues are shown. Scale bar: 100 μm. **c** Food intake ($n = 5$). **a, c** Data are presented as mean ± SEM. *$P < 0.05$, determined by two-tailed Student' s t-test. **b** Data are representative of three biologically independent replicates.

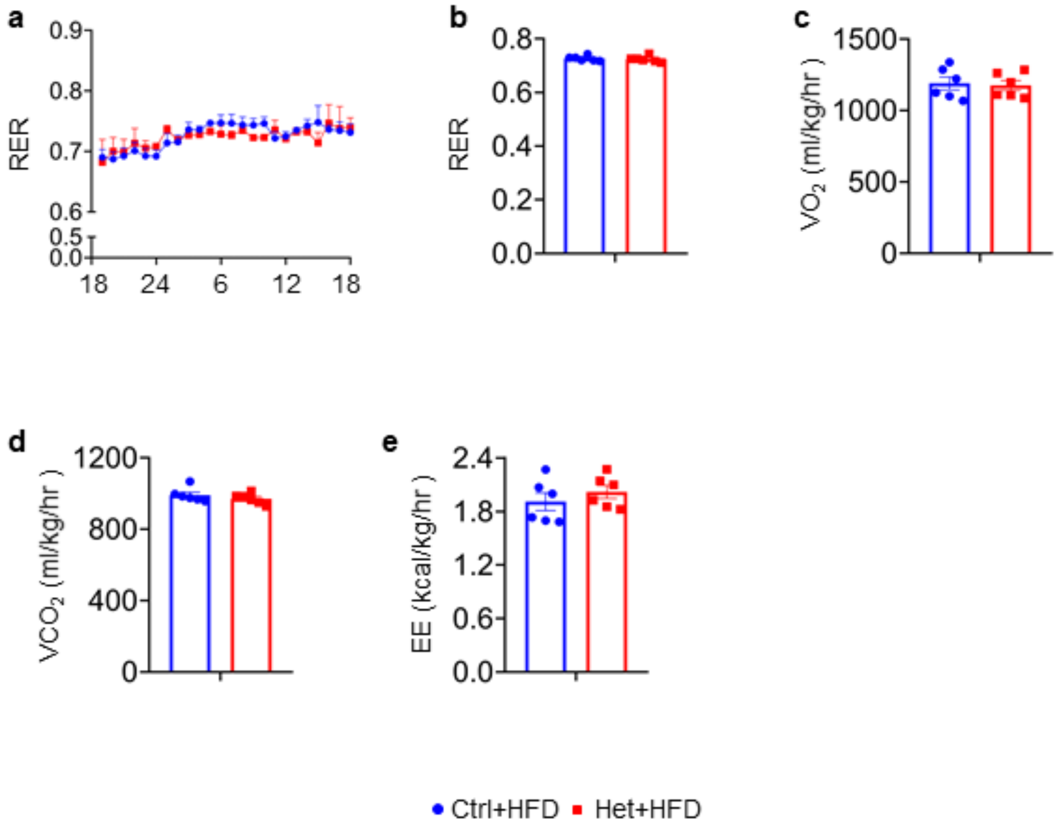

Supplementary Figure 4. Effects of Kindlin-2 haploinsufficiency on the basal metabolism and energy expenditure in mice. After 12-weeks HFD challenge, control and Het mice were individually housed in metabolic cages to measure the respiratory exchange rate (RER) **a**, **b** oxygen consumption rate (VO_2) **c** carbon dioxide production rate (VCO_2) **d**, and energy expenditure (EE) **e** ($n = 6$). **a-e** Data are presented as mean ± SEM.

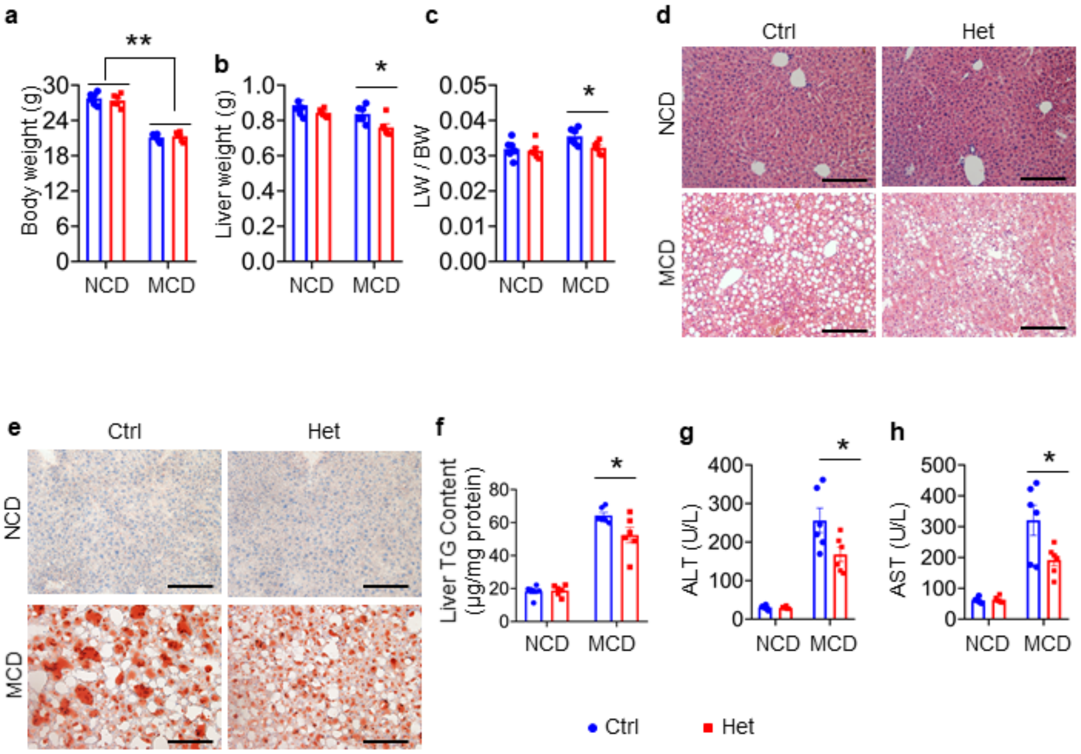

Supplementary Figure 5. Hepatic Kindlin-2 insufficiency ameliorates MCD-induced steatosis. a Body weight. Eight-week-old control and Kindlin-2 Het male mice were fed with NCD or MCD for 3 weeks. (*n* = 6) **b** Liver weight. (*n* = 6) **c** Liver/body weight ratio. **d** H/E staining of liver sections of the indicated groups, Scale bars,100 µm. **e** Oil Red O staining of liver sections of the indicated groups, Scale bars,100 µm. **f** Liver TG content. (*n* = 6) **g**, **h** Serum ALT and AST levels. (*n* = 6). **a, b, c, f, g, h** Data are presented as mean ± SEM. **P* < 0.05, **P < 0.01, determined by two-tailed Student's *t*-test. **d, e** Data are representative of three biologically independent replicates.

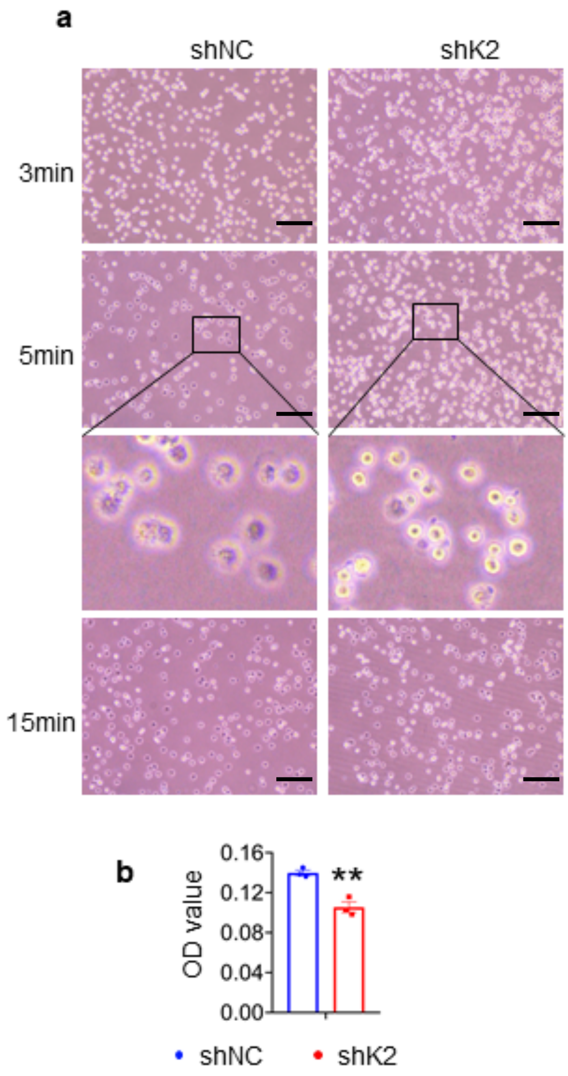

Supplementary Figure 6. Kindlin-2 KD impedes cell adhesion and proliferation.

a Cell adhesion. Huh7 cells transfected with control shRNA (sh-NC) or Kindlin-2 shRNAs (sh-K2). 96 h later, cells were seeded and recorded for cell adhesion by microscope. Scale bar: 100 μm. **b** CCK8 assay. Huh7 cells transfected with sh-NC or sh-K2. 96 h later, cells were seeded in a 96-well plate at a density of 2000 cells per well. After 24 h, 10 μl of CCK-8 reagent was added to each well and then cultured for 2 h. The absorbance was measured at 450 nm ($n = 3$). **b** Data are presented as mean ± SEM. $^{**}P < 0.01$, determined by two-tailed Student's t-test. **a** Data are representative of three biologically independent replicates.

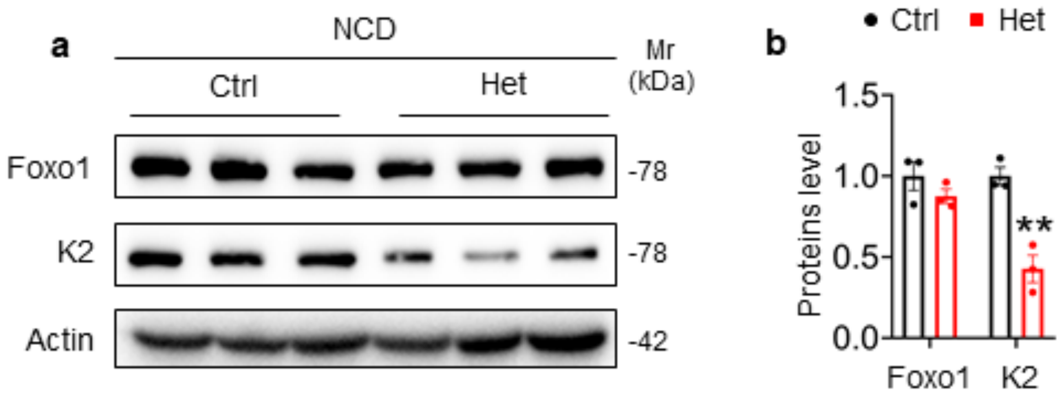

Supplementary Figure 7. Effect of Kindlin-2 haploinsufficiency on the level of Foxo1 protein in livers of mice under NCD-feeding condition. a Western blotting. The levels of Kindlin-2 and Foxo1 proteins in livers of control and Het mice fed on NCD for 12 weeks were determined by western blotting. 20 µg of protein extracts was used for western blotting from each sample. **b** Quantification of (**a**) ($n = 3$). **a. b** Data are presented as mean ± SEM. $^{**}P < 0.01$ vs Ctrl, determined by two-tailed Student's *t*-test. **a** Data are representative of three biologically independent replicates.

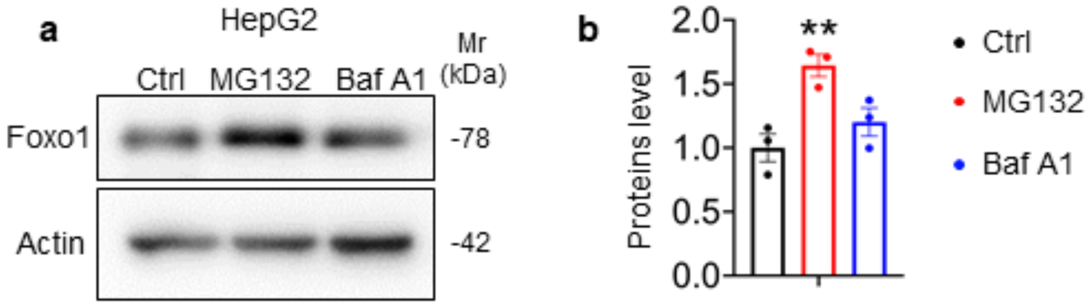

Supplementary Figure 8. Effect of MG132 and BafA1 on Foxo1 protein expression in HepG2 cells. **a** Western blotting. At 24 h after seeding, cells were treated with MG132 (10 μM) for 6 h or Baf A1 (10 nM) for 12 h, followed by western blotting using a Foxo1 antibody. Actin was used as a loading control. **b** Quantification of (**a**) from three independent experiments. **b** Data are presented as mean ± SEM. ***P* < 0.01 determined by two-tailed Student's *t*-test. **a** Data are representative of three biologically independent replicates.

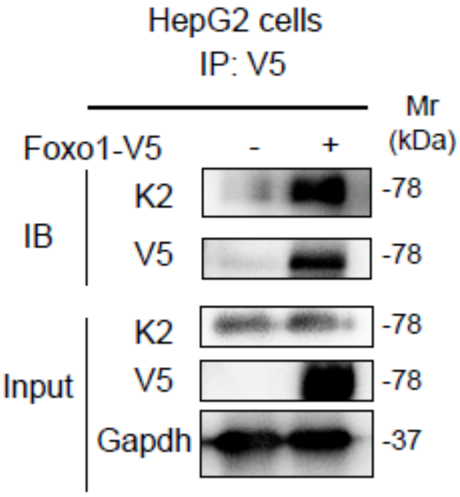

Supplementary Figure 9. Kindlin-2 interacts with Foxo1. HepG2 cells were transiently transfected with V5-tagged Foxo1 and the cell lysates were used for IP and IB with the antibodies as indicated. 200 µg of whole cell extracts from each group were used for the IP assays. Immunoprecipitates were resuspended in 50 µl buffer. 15 µl from each sample was loaded for SDS-PAGE, followed by western blotting analyses. Protein extracts (20 µg) were used for western blotting from each input. Gapdh was used as a loading control. Data are representative of three biologically independent replicates.

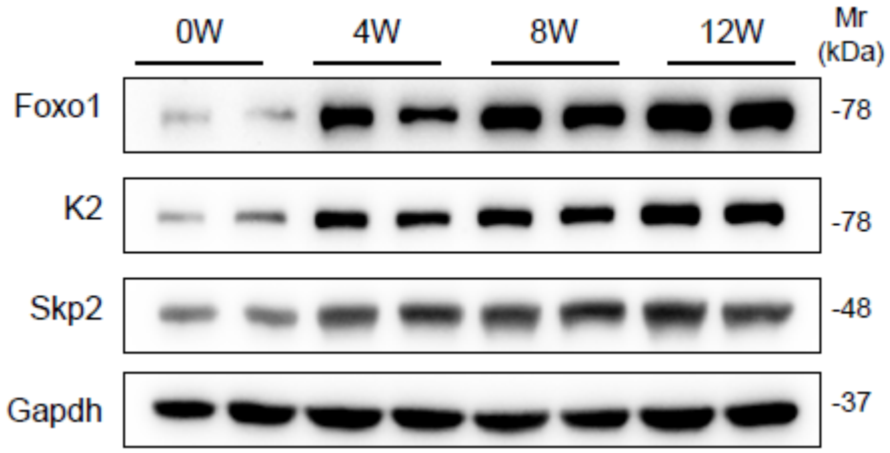

Supplementary Figure 10. Six-week-old C57BL/6 male mice were fed with HFD for 0, 4, 8 and 12 weeks. Whole cell extracts were prepared from liver tissues and subjected to western blotting. 20 μg protein extracts were used for western blotting from each sample. Data are representative of three biologically independent replicates.

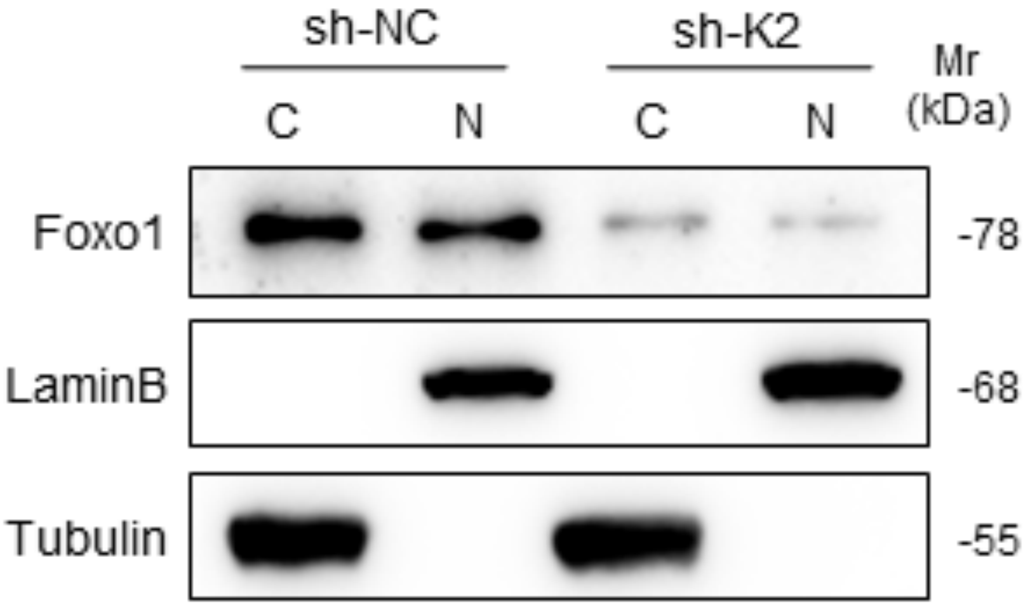

Supplementary Figure 11. Huh7 cells were infected with lentivirus expressing with control shRNA (sh-NC) and Kindlin-2 shRNAs (sh-K2). 96h later, cytoplasmic (C) and nuclear (N) extracts were prepared. 20 μg of protein extracts was used for western blotting from each group. Data is representative of three biologically independent replicates.

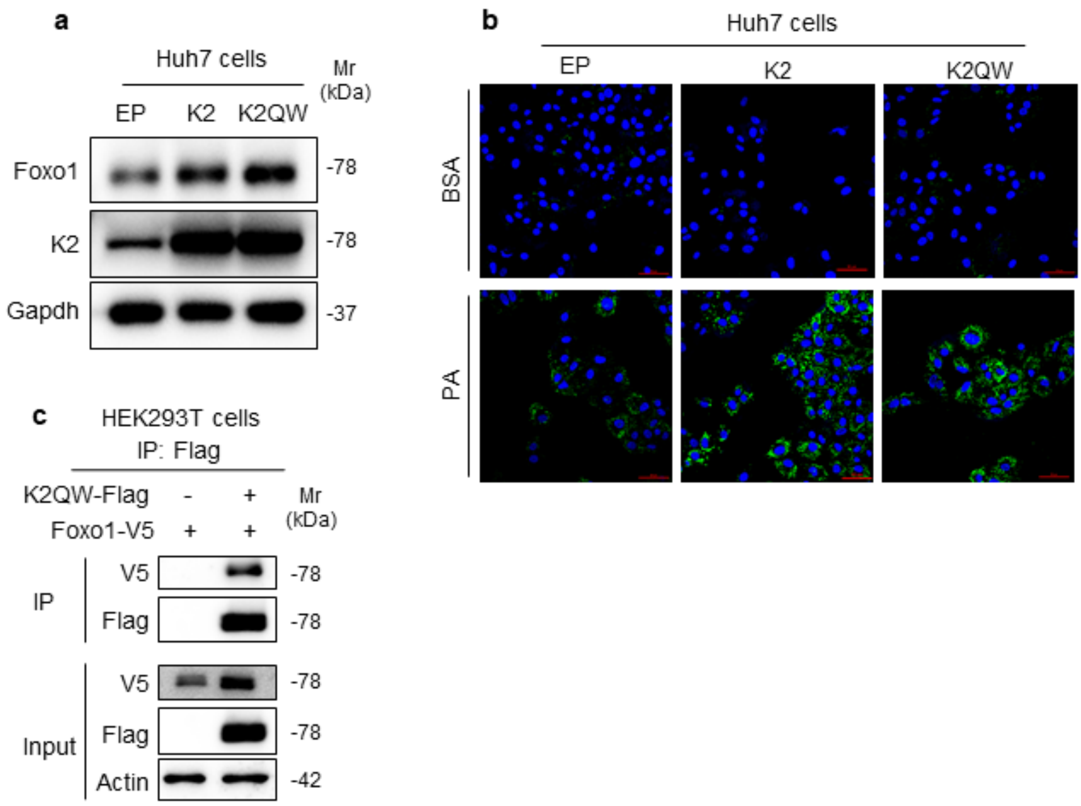

Supplementary Figure 12. Kindlin-2 regulates Foxo1 expression and lipid accumulation independent of integrin activation. (**a**) Huh7 cells were infected with empty vector lentivirus (EP), Kindlin-2 lentiviruses (K2) and an integrin-binding defective lentivirus (K2-QW). 96h later, cytoplasmic (C) and nuclear (N) extracts were prepared. 20 μg of protein extracts was used for western blotting from each group. (**b**) Huh7 cells were infected with EP, K2 and K2QW lentiviruses. 96h later, cells were treated with BSA or PA (200 mM) for another 18hours, followed by Bodipy staining, Scale bars, 50 μm. (**c**) co-IP assays. Cell lysates from HEK293T cells transfected with V5-tagged Foxo1 with and without Flag-K2QW were used for immunoprecipitation (IP) and immunoblotting (IB) with the indicated antibodies. **a-c** Data are representative of three biologically independent replicates.

Supplementary Table 1. Antibody information

Name	Supplier	Cat no.	Application/Dilution
Kindlin-2	Merck Millipore	MAB2617	WB (1:1000); IHC (1:200)
Gapdh	ZSGB-BIO	TA-08	WB (1:5000)
Actin	ZSGB-BIO	TA-09	WB (1:5000)
Flag	ZSGB-BIO	TA-05	WB (1:5000)
HA	Cell Signaling Technology Inc	3724	WB (1:2000)
Myc	Cell Signaling Technology Inc	2272	WB (1:2000)
V5	Cell Signaling Technology Inc	13202	WB (1:2000)
Tubulin	Cell Signaling Technology Inc	2128	WB (1:5000)
Fak	Abcam	ab40794	WB (1:1000)
Ilk	BD	611802	WB (1:1000)
Foxo1	Cell Signaling Technology Inc	2880	WB (1:1000)
p-Foxo1	Cell Signaling Technology Inc	9461	WB (1:1000)
Skp2	Cell Signaling Technology Inc	4313	WB (1:1000)
Lamin B	EASYBIO	BE3191	WB (1:1000)
Ubiquitin	Cell Signaling Technology Inc	3936	WB (1:2000)

Supplementary Table 2. Primer information for mouse

Gene	Forward	Reverse
Fas	GCGATGAAGAGCATGGTTTAG	GGCTCAAGGGTTCCATGTT
Kindlin-2	TGGACGGGATAAGGATGCCA	TGACATCGAGTTTTTCCAC CAAC
Srebp1c	GGAGCCATGGATTGCACATT	GGCCCGGGAAGTCACTGT
Acc	GCGGCTACAGGGACTATACTG	CGGAAGTAAGAGCTACTA GCGG
Scd1	CTGTACGGGATCATACTGGTTC	GCCGTGCCTTGTAAGTTCT G
Col1α1	TAGGCCATTGTGTATGCAGC	ACATGTTCAGCTTTGTGGA CC
Tgfβ	GTGGAAATCAACGGGATCAG	ACTTCCAACCCAGGTCCTT C
Tnfα	CCACGTCGTAGCAAACCACC	GATAGCAAATCGGCTGACG G
Mcp1	CACTCACCTGCTGCTACTCA	GCTTGGTGACAAAAACTA CAGC
Il-6	CTCATTCTGCTCTGGAGCCC	CAACTGGATGGAAGTCTCT TGC
α-Sma	GCTACTTACCCTGACAGCGA	TCCTTGTTTGGGAAGCGA GT
Ctgf	GCCTACCGACTGGAAGACAC	TGACTAGGGGCAGAGGAT GT
Foxo1	AAGAGTTAGTGAGCAGGCTAC AT	TTCCCAATGGCACAGTCCT T
Pparγ	GACGCGGAAGAAGAGACCTG	TCACCGCTTCTTTCAAATC TTGT
Gapdh	TTTCTTCTTGCCTTGGGAGA	AGTTCCGCACTTCATTCAG G

Supplementary Table 3. Primer information for humans

Gene	Forward	Reverse
FAS	CAACCTCTCCCAGGTATGCG	ACCCTTCAATCCCGTTGCAT
KINDLIN-2	GACCATGGCGGACAGTTCTT	TTCTCGCTGTTATCTGCTTGT
FOXO1	GAGGGTTAGTGAGCAGGTTACA	TGCTGCCAAGTCTGACGAAA
GAPDH	TCGGAGTCAACGGATTTGGT	TTCCCGTTCTCAGCCTTGAC

Supplementary Table 4. Clinical information of subjects included in this study

Characteristics	Normal (*n*=6)	NAFLD (*n*=8)	*P* value
Age (years)	31.85±7.9	40±10.9	0.337939
BMI (kg/m^2)	23.125±0.9	30.96±2.6	0.000327
ALT (IU/L)	22.35±3.3	31.61±4.0	0.00456
AST (IU/L)	27.675±5.0	49.5±11.1	0.006467
Triglycerides (mg/dL)	108.35±7.8	150.2±26.1	0.016852
Fasting blood glucose (mmol/L)	5.325±0.4	8.225±1.4	0.003765
Steatosis grade (0/1/2/3)	0	1.375±0.5	0.00041
Lobular inflammation (0/1/2/3)	0	1.25±0.4	0.000363
Ballooning (0/1/2)	0	1.25±0.4	0.000363

Abbreviations: NAFLD, nonalcoholic fatty liver disease; BMI, body mass index; ALT, alanine aminotransferase; AST, aspartate aminotransferase. The data are expressed as the means ± SDs or numbers (percentages). The statistical analyses (*P* value) were performed by comparing Normal vs. NAFLD by two-sided unpaired Student's t-tests.
