# Supplementary material for: Kindlin-2 haploinsufficiency protects against fatty liver by targeting Foxo1 in mice
Source: Nat Commun. 2022 Feb 23;13:1025. doi: 10.1038/s41467-022-28692-z (PMC8866405; doi:10.1038/s41467-022-28692-z)
Supplement: Supplementary file 3 — Reporting Summary [file 41467_2022_28692_MOESM3_ESM.pdf]

nature portfolio

Corresponding author(s): Guozhi Xiao

Last updated by author(s): Dec 20, 2021

Reporting Summary

Nature Portfolio wishes to improve the reproducibility of the work that we publish. This form provides structure for consistency and transparency in reporting. For further information on Nature Portfolio policies, see our Editorial Policies and the Editorial Policy Checklist.

Statistics

For all statistical analyses, confirm that the following items are present in the figure legend, table legend, main text, or Methods section.

n/a	Confirmed	
☐	☒	The exact sample size (*n*) for each experimental group/condition, given as a discrete number and unit of measurement
☐	☒	A statement on whether measurements were taken from distinct samples or whether the same sample was measured repeatedly
☐	☒	The statistical test(s) used AND whether they are one- or two-sided<br>*Only common tests should be described solely by name; describe more complex techniques in the Methods section.*
☐	☒	A description of all covariates tested
☐	☒	A description of any assumptions or corrections, such as tests of normality and adjustment for multiple comparisons
☐	☒	A full description of the statistical parameters including central tendency (e.g. means) or other basic estimates (e.g. regression coefficient) AND variation (e.g. standard deviation) or associated estimates of uncertainty (e.g. confidence intervals)
☐	☒	For null hypothesis testing, the test statistic (e.g. *F*, *t*, *r*) with confidence intervals, effect sizes, degrees of freedom and *P* value noted<br>*Give P values as exact values whenever suitable.*
☒	☐	For Bayesian analysis, information on the choice of priors and Markov chain Monte Carlo settings
☒	☐	For hierarchical and complex designs, identification of the appropriate level for tests and full reporting of outcomes
☒	☐	Estimates of effect sizes (e.g. Cohen's *d*, Pearson's *r*), indicating how they were calculated

Our web collection on statistics for biologists contains articles on many of the points above.

Software and code

Policy information about availability of computer code

Data collection	Nikon A1R Confocal Laser Scanning Microscope System used for immunofluorescence microscopy. cellSens Standard software (XV 3.17) used for collecting images of histology staining. ABI 7500 software used for collecting qPCR data.
Data analysis	NIS-Elements Viewer software (v. 5.21.00) used for analyzing confocal images. Graphpad Prism 8.0 used for graphing data and statistical analysis, Image J V1.52 used for analyzing western blot images.

For manuscripts utilizing custom algorithms or software that are central to the research but not yet described in published literature, software must be made available to editors and reviewers. We strongly encourage code deposition in a community repository (e.g. GitHub). See the Nature Portfolio guidelines for submitting code & software for further information.

Data

Policy information about availability of data

All manuscripts must include a data availability statement. This statement should provide the following information, where applicable:

- Accession codes, unique identifiers, or web links for publicly available datasets
- A description of any restrictions on data availability
- For clinical datasets or third party data, please ensure that the statement adheres to our policy

All data are available within the Article or Supplementary Information. Source data are provided with this paper. All data are also available from the corresponding author upon reasonable request.

Field-specific reporting

Please select the one below that is the best fit for your research. If you are not sure, read the appropriate sections before making your selection.

☒ Life sciences ☐ Behavioural & social sciences ☐ Ecological, evolutionary & environmental sciences

For a reference copy of the document with all sections, see nature.com/documents/nr-reporting-summary-flat.pdf

Life sciences study design

All studies must disclose on these points even when the disclosure is negative.

Sample size	No statistical method was used to predetermine sample size. Sample size was determined based on our own preliminary experiments and relevant literature in the field (PMID: 30033199; PMID: 34083525). Statistical significanse was obtained with these sample size. For animal study, to minimize use of animals, only male mice were used for experiments in this study. We used similar sample sizes for different animal groups (e.g. n=8 for control and KO groups; n=5 for shNC and shK2 groups; n=6 for AAV8-GFP and AAV8-K2 groups). For human data, n=4-6 for normal liver and n=6-8 for fatty liver were used.
Data exclusions	We excluded animals which developed abnormal disease or other diseases. The exclusion was made before group randomization, experimental intervention and data collection.
Replication	Experimental findings were reliably reproduced. The number of independent biologic replicates is indicated in each Figure Legend.
Randomization	Mice with different genotypes were randomly divided into different experimental groups. Cells were grown under the same conditions and randomly allocated into different treatment groups without any bias.
Blinding	The investigators were blinded to group allocation during data collection and analysis.

Reporting for specific materials, systems and methods

We require information from authors about some types of materials, experimental systems and methods used in many studies. Here, indicate whether each material, system or method listed is relevant to your study. If you are not sure if a list item applies to your research, read the appropriate section before selecting a response.

Materials & experimental systems

n/a	Involved in the study	
☐	☒	Antibodies
☐	☒	Eukaryotic cell lines
☒	☐	Palaeontology and archaeology
☐	☒	Animals and other organisms
☐	☒	Human research participants
☒	☐	Clinical data
☒	☐	Dual use research of concern

Methods

n/a	Involved in the study	
☒	☐	ChIP-seq
☒	☐	Flow cytometry
☒	☐	MRI-based neuroimaging

Antibodies

Antibodies used	Anti-Kindlin-2 antibody (Merck Millipore MAB2617 WB 1:1000, IHC 1:200), Anti-Gapdh antibody (ZSGB-BIO TA-08 WB 1:5000), Anti-Actin antibody (ZSGB-BIO TA-09 WB 1:5000), Anti-Flag antibody (ZSGB-BIO TA-05 WB 1:5000), Anti-HA antibody (Cell Signaling Technology 3724 WB 1:2000), Anti-Myc antibody (Cell Signaling Technology 2272 WB 1:2000), Anti-V5 antibody (Cell Signaling Technology 13202 WB 1:2000), Anti-Tubulin antibody (Cell Signaling Technology 2128 WB 1:5000), Anti-Fak antibody (Abcam ab40794 WB 1:1000), Anti-Ilk antibody (BD 611802 WB 1:1000), Anti-Foxo1 antibody(Cell Signaling Technology 2880 WB 1:1000, IF 1:200), Anti-p-Foxo1 antibody(Cell Signaling Technology 9461 WB 1:1000), Anti-Skp2 antibody (Cell Signaling Technology 4313 WB 1:1000), Anti-Lamin B antibody (EASYBIO BE3191 WB 1:1000), Anti-Ubiquitin antibody(Cell Signaling Technology 3936 WB 1:2000)<br>Secondary antibodies:<br>Goat anti-Rabbit IgG Alexa Fluor 568 (Invitrogen A-11036 IF1:500), Goat anti-Mouse IgG, Alexa Fluor 488 (Invitrogen A-11001 IF1:500), Goat anti-Mouse IgG(H+L) HRP-linked Antibody (ZSGB-BIO ZB-2305 WB 1:5000), Goat anti-Rabbit IgG(H+L) HRP-linked Antibody (ZSGB-BIO ZB-2301 WB 1:5000)
Validation	All antibodies were commercially validated for the application used. In addition, all antibodies used for western blotting showed bands at the expected sizes.<br>Anti-Foxo1 antibody (https://www.cellsignal.cn/products/primary-antibodies/foxo1-c29h4-rabbit-mab/2880?site-search-type=Products&N=4294956287&Ntt=foxo1&fromPage=plp)<br>Anti-Kindlin-2 antibody (https://www.sigmaaldrich.cn/CN/zh/product/mm/mab2617?context=product)<br>Anti-Gapdh antibody (http://www.zsbio.com/product/TA-08)

Anti-Actin antibody (http://www.zsbio.com/product/TA-09)
Anti-Flag antibody (http://www.zsbio.com/product/TA-05)
Anti-HA antibody (https://www.cellsignal.cn/products/primary-antibodies/ha-tag-c29f4-rabbit-mab/3724?site-search-type=Products&N=4294956287&Ntt=3724&fromPage=plp&_requestid=431506)
Anti-Myc antibody (https://www.cellsignal.cn/products/primary-antibodies/myc-tag-antibody/2272?site-search-type=Products&N=4294956287&Ntt=2272&fromPage=plp&_requestid=431560)
Anti-V5 antibody (https://www.cellsignal.cn/products/primary-antibodies/v5-tag-d3h8q-rabbit-mab/13202?site-search-type=Products&N=4294956287&Ntt=13202&fromPage=plp&_requestid=431611)
Anti-Tubulin antibody (https://www.cellsignal.cn/products/primary-antibodies/b-tubulin-9f3-rabbit-mab/2128?site-search-type=Products&N=4294956287&Ntt=2128&fromPage=plp&_requestid=431687)
Anti-Fak antibody (https://www.abcam.cn/fak-antibody-ep695y-ab40794.html)
Anti-Ilk antibody (https://www.labome.com/product/BD-Biosciences/611802.html)
Anti-Foxo1 antibody (https://www.cellsignal.cn/products/primary-antibodies/foxo1-c29h4-rabbit-mab/2880?site-search-type=Products&N=4294956287&Ntt=2880&fromPage=plp&_requestid=432249)
Anti-p-Foxo1 antibody (https://www.cellsignal.cn/products/primary-antibodies/phospho-foxo1-ser256-antibody/9461?site-search-type=Products&N=4294956287&Ntt=9461&fromPage=plp&_requestid=432296)
Anti-Skp2 antibody (https://www.cellsignal.cn/products/primary-antibodies/skp2-l70-antibody/4313?site-search-type=Products&N=4294956287&Ntt=4313&fromPage=plp&_requestid=432422)
Anti-Lamin B antibody (http://www.bioeasytech.com/home/product/article/id/185/sear/lam.html
Anti-Ubiquitin antibody https://www.cellsignal.cn/products/primary-antibodies/ubiquitin-p4d1-mouse-mab/3936?site-search-type=Products&N=4294956287&Ntt=ub&fromPage=plp)
Secondary antibodies:
Goat anti-Mouse IgG(H+L) HRP-linked Antibody (http://www.zsbio.com/product/ZB-2305)
Goat anti-Rabbit IgG(H+L) HRP-linked Antibody (http://www.zsbio.com/product/ZB-2301)
Goat anti-Rabbit IgG Secondary Antibody, Alexa Fluor 568 (https://www.thermofisher.cn/cn/zh/antibody/product/Goat-anti-Rabbit-IgG-H-L-Highly-Cross-Adsorbed-Secondary-Antibody-Polyclonal/A-11036)
Goat anti-Mouse IgG Secondary Antibody, Alexa Fluor 488 (https://www.thermofisher.cn/cn/zh/antibody/product/Goat-anti-Mouse-IgG-H-L-Cross-Adsorbed-Secondary-Antibody-Polyclonal/A-11001)

Eukaryotic cell lines

Policy information about cell lines

Cell line source(s)	HepG2 and Huh7 cells were purchased from the Cell Bank of the Chinese Academy of Sciences. HEK293T cell was obtained from ATCC.
Authentication	The cell line was not authenticated.
Mycoplasma contamination	The results of mycoplasma contaimination test of HepG2, Huh7 and HEK293T cell and the cell culture facility were negative.
Commonly misidentified lines (See ICLAC register)	No commonly misidentified cell lines were used.

Animals and other organisms

Policy information about studies involving animals; ARRIVE guidelines recommended for reporting animal research

Laboratory animals	The animal use and the experimental protocols were reviewed and approved by the Animal Care Committee of the Southern University of Science and Technology in accordance with the Institutional Animal Care and Use Committee guidelines. Male Kindlin-2f/+ mice; Alb-Cre;Kindlin-2f/+mice; C57BL6; ob/ob mice were monitored from birth to 5-month-old. The mice were sacrificed at indicated time in the figure legends. 8-week-old male Kindlin-2f/+mice; Alb-Cre;Kindlin-2f/+ mice and ob/ob mice were treatment with AAV-Foxo1/AAV-GFP or AAV-shk2/AAV-shNC respectively. All mice were given food and water ad libitum and housed in temperature-, moisture-, and light-controlled (12h light/dark cycle).
Wild animals	Wild animals were not involved.
Field-collected samples	Field-collected samples were not involved.
Ethics oversight	All research protocols in this study were approved by the Institutional Animal Care and Use Committees (IACUC) of Southern University of Science and Technology (Approval No: SUSTC-JY2019042) in accordance with the Institutional Animal Care and Use Committee guidelines. All efforts were made to reduce the number of animals used and to minimize animal suffering.

Note that full information on the approval of the study protocol must also be provided in the manuscript.

Human research participants

Policy information about studies involving human research participants

Population characteristics	Eight patients with NAFLD without obesity and 6 non-obese healthy comparison subjects with normal liver on MRI were included in the study. All the patients had no history of any hepatotoxic drugs, hormone replacement therapy, or herbal products and consumed no more than 20 g/day alcohol. The healthy control group had no illness, no usage of alcohol, drugs, or herbal substances, no history of previous liver diseases, and was negative for viral hepatitis serology tests and had normal liver. Clinical characteristics of the human research participants was provided in Supplementary Table 4.

Recruitment	All human blood samples and clinical information were obtained from the People's Hospital of Guiyang. Informed consent was obtained from participants included in this study. Six non-obese healthy subjects and 8 NAFLD without obesity subjects diagnosed with MRI were included in the study. These participants were recruited randomly from the physical examination population, so there was no potential self-selection bias or other biases that may be present and impact results.
Ethics oversight	Normal and NAFLD liver tissues were obtained from healthy individuals or patients with NAFLD undergoing biopsy or transplantation in the People's Hospital of Guiyang (Guiyang, China). Procedures were approved by the ethics committee of People's Hospital of Guiyang (IRB# 2019-202).

Note that full information on the approval of the study protocol must also be provided in the manuscript.
